# Invariant Natural Killer T Cells in Cancer Immunotherapy: Lipid-Based Modulation, Nanotechnology, and Translational Advances

**DOI:** 10.3390/ijms27062528

**Published:** 2026-03-10

**Authors:** Abdulaziz A. Aloliqi, Abdullah M. Alnuqaydan, Mohammad Alshebremi, Arif Khan, Masood Alam Khan

**Affiliations:** 1 Department of Basic Health Sciences, College of Applied Medical Sciences, Qassim University, Buraydah 51452, Saudi Arabia; aaalieky@qu.edu.sa (A.A.A.); ami.alnuqaydan@qu.edu.sa (A.M.A.); 4140@qu.edu.sa (A.K.); 2 Department of Medical Laboratories, College of Applied Medical Sciences, Qassim University, Buraydah 51452, Saudi Arabia; m.alshebremi@qu.edu.sa

**Keywords:** cancer immunotherapy, iNKT cells, α-GalCer, nanoparticles, tumor microenvironment

## Abstract

Invariant natural killer T (iNKT) cells are a unique lymphocyte subset that bridge innate and adaptive immunity through recognition of glycolipid antigens presented by CD1d. Upon activation by ligands such as α-galactosylceramide (α-GalCer), iNKT cells rapidly secrete cytokines, including IFN-γ and TNF-α, thereby activating dendritic cells, natural killer (NK) cells, and cytotoxic T lymphocytes (CTLs) to promote antitumor immunity. Despite their therapeutic promise, clinical translation has been limited by rapid α-GalCer clearance, induction of iNKT cell anergy following repeated stimulation, and the immunosuppressive tumor microenvironment (TME). Recent advances in lipid-engineered nanoparticle systems offer solutions to these challenges by improving ligand stability, enhancing antigen-presenting cell targeting, and enabling controlled release that sustains Th1-biased activation while reducing anergy. Liposomal and polymer-based nano-formulations enhance bioavailability and promote more durable IFN-γ-mediated responses. In parallel, chimeric antigen receptor (CAR)-engineered iNKT cells provide antigen-specific tumor targeting while preserving intrinsic CD1d-restricted immunomodulatory functions, demonstrating encouraging safety and efficacy in early-phase studies. Combination strategies further strengthen iNKT-based immunotherapy. Integration with chemotherapy, immune checkpoint inhibitors such as anti-PD-1 and anti-CTLA-4, and cytokine support enhances effector activation, counteracts TME-induced suppression, and improves therapeutic outcomes. However, challenges remain, including optimization of dosing, control of off-target immune activation, scalable manufacturing, and long-term safety evaluation. Collectively, the convergence of nanotechnology, CAR engineering, and rational combination approaches establishes iNKT cell-based therapy as a promising next-generation immunotherapeutic strategy. Continued refinement of delivery systems, genetic engineering platforms, and translational protocols may enable durable immune reprogramming and improved clinical outcomes in resistant and immunosuppressive cancers.

## 1. Introduction

Cancer remains one of the leading causes of death worldwide, accounting for approximately 9.5 million deaths in 2018, according to the World Health Organization (WHO) [1]. Its high mortality rate is largely attributable to hallmark characteristics such as uncontrolled cellular proliferation, invasive growth, and metastatic dissemination, with metastasis alone responsible for nearly 90% of cancer-related deaths [2]. While localized tumors can often be treated effectively with surgery and radiotherapy, chemotherapy has historically been the cornerstone of treatment for advanced disease. However, its clinical utility is frequently limited by significant systemic toxicity and therapy-induced immunosuppression [3]. Over the past decade, immunotherapy has fundamentally reshaped the cancer treatment landscape. Immune checkpoint inhibitors (ICIs) targeting CTLA-4 and PD-1/PD-L1 pathways have demonstrated durable clinical responses and are now approved for multiple malignancies, serving as first-line therapies in cancers such as melanoma, non-small-cell lung cancer, renal cell carcinoma, and hepatocellular carcinoma [4]. In parallel, chimeric antigen receptor (CAR)-T cell therapies have achieved regulatory approval for several hematologic malignancies, underscoring the transformative potential of engineered cellular immunotherapy [5].

**Figure 1:**
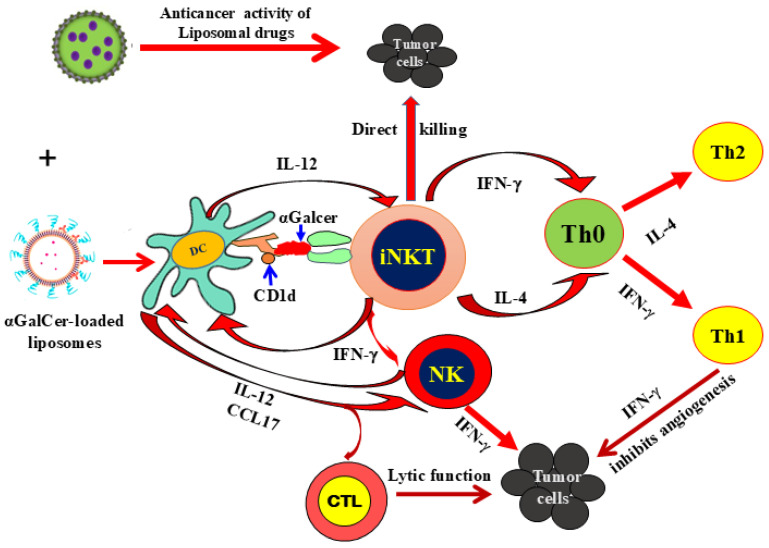
Antitumor and immunomodulatory functions of iNKT cells. iNKT cells exert antitumor effects through both direct cytotoxicity and indirect mechanisms by activating other immune effector cells, including natural killer (NK) cells and CTLs, thereby enhancing overall immune response against tumors.

Despite these advances, a substantial proportion of patients experience primary resistance or disease relapse, particularly in solid tumors, highlighting the need for complementary and more versatile immunotherapeutic strategies. In this context, invariant Natural Killer T (iNKT) cells have emerged as promising candidates. Positioned at the interface of innate and adaptive immunity, iNKT cells recognize glycolipid antigens presented by CD1d molecules on antigen-presenting cells [6,7]. Upon activation by ligands such as α-galactosylceramide (α-GalCer), they rapidly secrete large amounts of cytokines, including IFN-γ and TNF-α, which activate NK cells and cytotoxic T lymphocytes (CTLs), thereby promoting potent antitumor immune responses. At the same time, they can produce Th2 cytokines such as IL-4, contributing to immune regulation and homeostasis [8] (Figure 1). This unique capacity to orchestrate both pro-inflammatory and regulatory immune responses positions iNKT cells as attractive and versatile candidates for next-generation cancer immunotherapy.

Despite their considerable therapeutic promise, several challenges limit the clinical translation of iNKT cell-based immunotherapy. A major obstacle is the poor aqueous solubility and rapid systemic clearance of α-GalCer, which leads to only transient immune activation and suboptimal therapeutic durability [9]. Furthermore, repeated administration of soluble α-GalCer can induce iNKT cell anergy, a hyporesponsive state that significantly diminishes antitumor efficacy. This phenomenon is largely attributed to antigen presentation by non-professional antigen-presenting cells (APCs) that lack essential co-stimulatory signals required for sustained activation [10,11,12]. In addition, many cancer patients exhibit reduced numbers and impaired functionality of circulating iNKT cells, further compromising therapeutic outcomes [13].

To overcome these limitations, innovative strategies have been developed to enhance both iNKT cell activation and delivery efficiency. Nanoparticle-based delivery systems, including liposomal formulations and biodegradable polymeric carriers, have demonstrated improved ligand stability, preferential targeting to professional APCs, and sustained iNKT activation within the tumor microenvironment (TME) [14,15,16]. These platforms enhance antigen presentation while reducing the risk of overstimulation-induced anergy. In parallel, advances in cellular engineering have enabled the development of chimeric antigen receptor-modified iNKT cells (CAR-iNKT), which combine intrinsic CD1d-restricted immunoregulatory functions with antigen-specific tumor targeting. CAR-iNKT cells exhibit enhanced tumor specificity and improved resistance to immunosuppressive signals within the TME [17].

Combination immunotherapy approaches further amplify the therapeutic potential of iNKT-based strategies. For example, co-administration of α-GalCer-pulsed dendritic cells (DCs) with immune checkpoint inhibitors, such as anti-PD-1 or anti-CTLA-4 antibodies, has shown synergistic effects in preclinical models, enhancing iNKT activation, reducing immune exhaustion, and prolonging survival [18]. Similarly, cytokine adjuvants such as IL-12 and IL-18 have been employed to potentiate antitumor responses by augmenting NK cell and CTL activation [19]. Although these approaches represent substantial progress, important translational challenges remain. Optimization of dosing strategies, mitigation of off-target immune activation, and establishment of scalable, standardized manufacturing processes are critical for clinical implementation. Future research should focus on refining nano-delivery systems, developing off-the-shelf allogeneic iNKT cell products, and designing rational combination regimens capable of overcoming tumor-induced immune suppression and broadening the clinical applicability of iNKT-based immunotherapy.

## 2. Immunoediting and Tumor Evolution

The relationship between cancer and the immune system is complex and dynamic. Early observations by Rudolf Virchow linked chronic inflammation to tumor development [20], a concept that later evolved into the framework of cancer immunoediting. Immunoediting describes how the immune system not only suppresses tumor growth but also shapes tumor evolution through three interconnected phases: elimination, equilibrium, and escape [21,22,23]. These phases reflect a continuous balance between immune surveillance and tumor adaptation, influenced by immune cell composition, antigenicity, inflammatory mediators, and the development of immunological memory. In the elimination phase, innate and adaptive immune mechanisms cooperate to detect and eradicate emerging malignant cells. Type I interferons activate DCs and promote CTL priming [24], while danger-associated molecular patterns (DAMPs) released from stressed or dying tumor cells enhance immune recruitment and activation [25]. Tumor-derived neoantigens presented via MHC class I molecules stimulate CD8^+^ T cells, which secrete IFN-γ to inhibit tumor proliferation, enhance antigen presentation, and promote apoptotic pathways in cancer cells [26]. Clinically, high infiltration of CD8^+^ T cells correlates with improved prognosis in melanoma, underscoring the functional relevance of this immune surveillance phase in human cancer [27].

If complete eradication fails, residual tumor cells may enter a state of immune-mediated dormancy known as the equilibrium phase. During this stage, continuous immune pressure, primarily from CTLs and helper T cells, restrains tumor progression without fully eliminating malignant cells. Experimental evidence from murine models shows that NK cell depletion permits methylcholanthrene-induced sarcoma development, highlighting the importance of ongoing immune containment [28,29]. Cytokine balance also plays a decisive role: IL-12 supports tumor control, whereas IL-23 promotes tumor progression [30]. Clinical observations in low-grade B-cell lymphoma and pediatric acute myeloid leukemia (AML) further support this concept, as prolonged remission has been associated with preserved cytotoxic T cell function [31,32]. These findings suggest that immune-mediated equilibrium can delay or prevent tumor outgrowth.

Ultimately, tumor variants may acquire adaptations that enable immune escape and clinically apparent disease. This escape phase is characterized by genetic and epigenetic alterations that impair immune recognition and foster an immunosuppressive TME. Downregulation of MHC class I molecules, often through β2-microglobulin loss, limits CD8^+^ T cell recognition [33]. In papillary thyroid carcinoma, reduced MHC class I expression correlates with diminished T cell infiltration, although restoration of antigen presentation can be achieved with IFN-γ or MEK1/2 inhibition [33]. Oncogenic signaling pathways such as STAT3 and Bcl-2 further promote tumor survival and immune suppression, as demonstrated in mammary tumor models [34]. In addition, upregulation of immune checkpoint molecules, particularly PD-L1, suppresses T cell activation via PD-1 signaling, driving functional exhaustion and facilitating immune evasion [35]. These mechanistic insights provided the foundation for the development of immune checkpoint blockade therapies aimed at restoring effective antitumor immunity.

## 3. Tumor Immune Evasion: Disrupting Recognition, Suppressing Response

**Figure 2:**
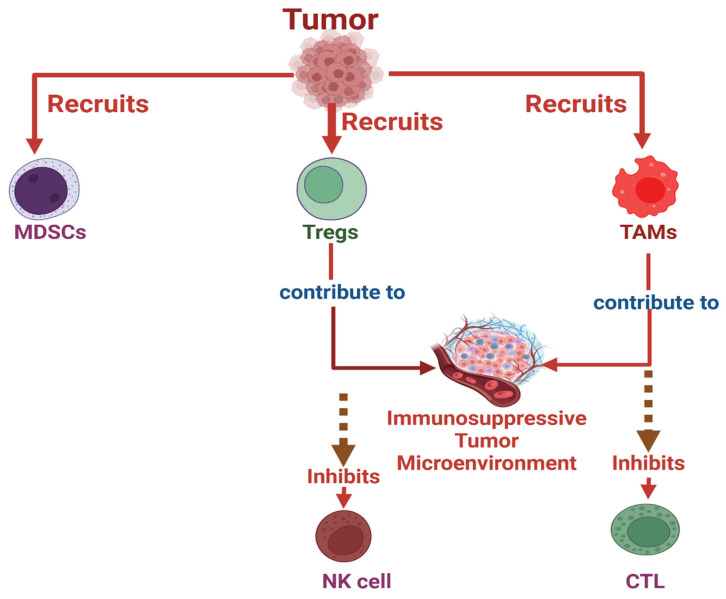
Tumor-driven recruitment of immunosuppressive cells and establishment of an inhibitory tumor microenvironment. Tumor cells promote immune evasion by actively recruiting immunosuppressive cell populations, including MDSCs, regulatory T cells (Tregs), and tumor-associated macrophages (TAMs). These cells contribute to the formation of suppressive TME that inhibits the cytotoxic functions of NK cells and CTLs, thereby weakening antitumor immune responses and facilitating tumor progression.

Despite the immune system’s intrinsic capacity to detect and eliminate malignant cells, tumors have evolved sophisticated immune evasion strategies that not only prevent immune recognition but also actively reshape the immune landscape to favor disease progression. These mechanisms operate at multiple levels, including disruption of antigen presentation, exploitation of immune checkpoint pathways, recruitment of suppressive immune cells, and modulation of cytokine networks. Collectively, these processes establish a hostile tumor microenvironment (TME) that impairs both innate and adaptive immune responses (Figure 2).

One prominent mechanism of immune escape involves the disruption of antigen presentation. Tumor cells frequently downregulate β2-microglobulin (β2m), a critical structural component of both MHC class I and CD1d molecules. Loss of β2m impairs antigen presentation, thereby reducing tumor visibility to cytotoxic T lymphocytes (CTLs) and invariant natural killer T (iNKT) cells [36]. In addition, tumors exploit epigenetic silencing mechanisms to suppress genes involved in antigen processing, including transporters associated with antigen processing (TAP) and endoplasmic reticulum aminopeptidases (ERAPs). This compromises peptide loading onto MHC class I molecules and limits effective antigen display on the tumor surface, allowing malignant cells to evade immune detection [37,38]. Beyond antigen presentation defects, tumors further suppress immune responses by exploiting inhibitory checkpoint pathways. Many tumor cells upregulate ligands such as PD-L1, CD200, and HLA-E, which engage inhibitory receptors including PD-1, CD200R, and NKG2A on T cells and NK cells. These interactions attenuate effector functions and induce functional exhaustion, thereby promoting immune tolerance and sustained tumor survival [39]. This checkpoint-driven immune suppression forms the biological basis for immune checkpoint blockade (ICB) therapies.

**Figure 3:**
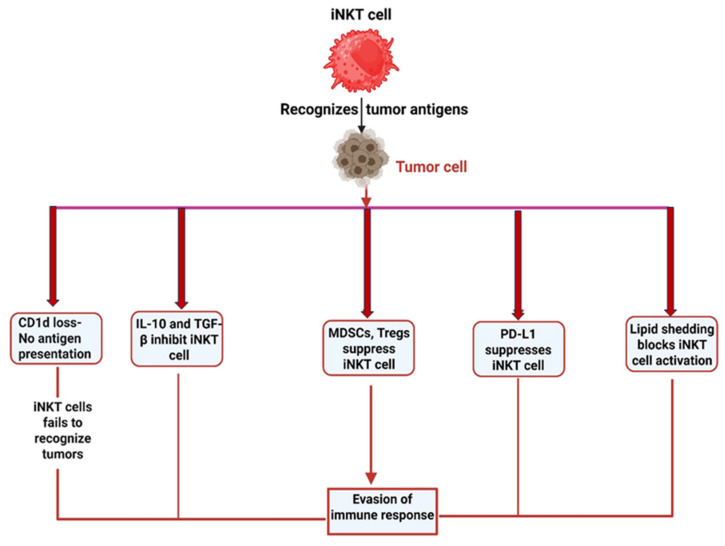
Evasion of iNKT Cell Antitumor Activity by Tumor Cells. Tumor cells evade iNKT cell-mediated immune responses through multiple mechanisms, including CD1d downregulation, secretion of IL-10 and TGF-β, recruitment of regulatory cells (MDSCs, Tregs), upregulation of PD-L1, and shedding of lipids to block iNKT activation.

Tumors also actively reprogram the TME to reinforce immune suppression at its source. They recruit immunosuppressive cell populations such as myeloid-derived suppressor cells (MDSCs), regulatory T cells (Tregs), and tumor-associated macrophages (TAMs). These cells secrete inhibitory cytokines, including TGF-β, IL-10, and VEGF, which suppress CTL, NK cell, and iNKT cell activation, inhibit antigen presentation, and promote angiogenesis, collectively fostering tumor progression (Figure 2 and Figure 3) [39,40,41]. Importantly, tumors deploy additional strategies to specifically undermine iNKT cell function. Unlike conventional T or NK cells, iNKT cells recognize lipid antigens presented by CD1d. Tumor cells can evade this recognition by downregulating CD1d expression or interfering with glycolipid antigen presentation. For example, breast cancer cells have been shown to reduce CD1d expression, decreasing their susceptibility to iNKT-mediated cytotoxicity. Similarly, ovarian cancer and murine lymphoma cells can shed inhibitory glycolipids that disrupt CD1d–antigen complexes, thereby preventing effective iNKT activation (Figure 3) [36,42]. These findings underscore the tumor’s capacity to selectively target non-classical immune pathways critical for iNKT-mediated antitumor immunity.

Interestingly, iNKT cells are not only susceptible to suppression but also possess the capacity to counteract immunosuppressive networks. During influenza A infection, iNKT cells have been shown to reduce MDSC-mediated suppression, restoring immune balance under inhibitory conditions [43,44,45]. This immunoregulatory capability suggests that therapeutic reactivation of iNKT cells may help reverse tumor-induced immune suppression, particularly in TMEs enriched with MDSCs. Nevertheless, in cancer patients, iNKT cell dysfunction is commonly observed at both quantitative and functional levels. Several studies have reported significantly reduced frequencies of circulating Vα24^+^ iNKT cells in patients with hematologic malignancies and solid tumors, independent of tumor burden [46,47]. Moreover, patients with myelodysplastic syndrome (MDS) often exhibit a marked deficiency in IFN-γ-producing iNKT cells, reflecting impaired effector function [48]. This dual impairment in number and functionality may represent an adaptive tumor strategy to evade immune destruction. Encouragingly, clinical interventions aimed at restoring iNKT cell activity, such as α-GalCer stimulation, DC-based vaccination, or adoptive iNKT cell transfer, have demonstrated enhanced immune responses and improved clinical outcomes in selected patient populations [49].

## 4. Reprogramming the TME: The Central Role of iNKT Cells in Immune Restoration

**Figure 4:**
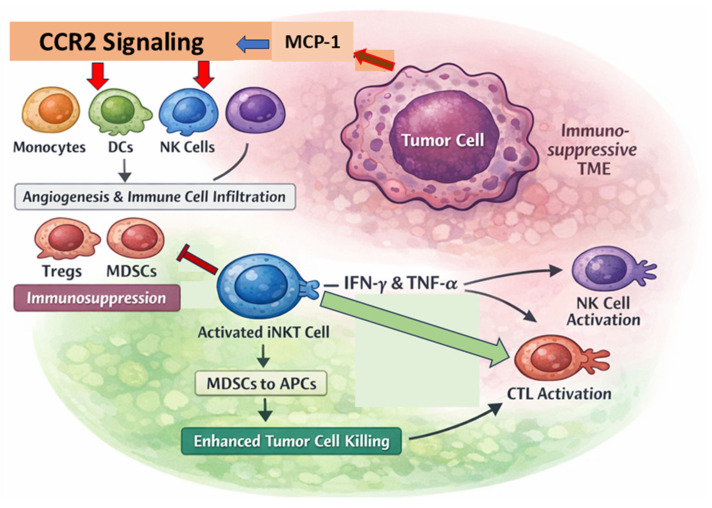
Interaction of iNKT cells with TME. Tumor-derived MCP-1 recruits monocytes, DCs, and NK cells through CCR2 signaling, promoting angiogenesis and immune cell infiltration but also fostering immunosuppression through Tregs and MDSCs. Activated iNKT cells release IFN-γ and TNF-α, which convert MDSCs into APCs. These cytokines also activate NK cells and CTLs, strengthening tumor cell killing and shifting the TME from an immunosuppressive to an immunostimulatory state.

Tumor progression is not solely driven by intrinsic genetic mutations but is heavily influenced by the TME, a dynamic and complex niche composed of malignant cells, stromal components, immune infiltrates, and extracellular matrix structures. Far from being a passive bystander, the TME actively facilitates tumor growth, immune evasion, and metastasis by reshaping both its biochemical and structural landscape (Figure 4).

### 4.1. Tumor-Driven Remodeling of the TME

Tumors modulate their surroundings by releasing chemokines that orchestrate immune cell infiltration. A key player is monocyte chemoattractant protein-1 (MCP-1), which binds to CCR2 on immune cells, recruiting monocytes, memory T cells, NK cells, and DCs into the tumor bed (Figure 4) [50]. While such recruitment may appear beneficial for immune surveillance, tumors subvert this process by polarizing infiltrating cells into immunosuppressive phenotypes, such as TAMs, Tregs, and MDSCs, which sustain an immune-tolerant and tumor-permissive environment. The biochemical profile of the TME is equally hostile to antitumor immunity. It is enriched with immunosuppressive cytokines like IL-10, TGF-β, and VEGF, which blunt effector T and NK cell responses, reduce antigen presentation, and suppress co-stimulatory signaling [50,51].

### 4.2. iNKT Cells: Reshaping the Immunosuppressive Landscape

Within this hostile microenvironment, iNKT cells emerge as potent immunomodulators capable of reconditioning the TME into an immunostimulatory niche [52]. Unlike conventional T cells, iNKT cells are resilient to many suppressive cues in the TME and can initiate broad immune activation through rapid cytokine release and cell–cell interactions. One of the critical functions of iNKT cells is their ability to neutralize MDSCs, which are key suppressors of T and NK cell cytotoxicity [53]. Preclinical studies have shown that IFN-γ secreted by iNKT cells reprograms MDSCs into APCs, thereby reversing their suppressive role and enhancing antitumor immunity [54]. iNKT-derived TNF-α further disrupts the cytokine environment favorable to tumor survival, restoring immune competence in the TME [55]. Importantly, activated iNKT cells also counteract tumor-mediated antigen presentation deficits. Tumors often downregulate MHC class I and CD1d molecules to escape detection. However, iNKT cells can activate DCs, enhancing their expression of co-stimulatory molecules and MHC/CD1d complexes, thereby improving antigen presentation and facilitating the priming of effector T cells [56].

### 4.3. Amplifying the Immune Orchestra: iNKT Cells as Master Coordinators

Beyond direct immunomodulation, iNKT cells function as central conductors of the immune orchestra. Through the secretion of IL-12, IFN-γ, and GM-CSF, they promote the activation and recruitment of cytotoxic lymphocytes, including CD8^+^ T cells and NK cells, which mediate tumor cell lysis through perforin–granzyme and Fas-FasL pathways (Figure 4) [57]. Additionally, iNKT cells interact with DCs to upregulate antigen presentation and co-stimulatory signals, boosting the priming and expansion of tumor-specific T cells and bridging the gap between innate and adaptive immunity [58]. They also attenuate the suppressive influence of TAMs and Tregs within the TME. By limiting the abundance or function of these suppressor cells, iNKT cells relieve the brakes on effector immunity, unleashing a more potent and coordinated attack on tumor cells [59].

## 5. iNKT Cells in Cancer Immunity: Subset Biology, Therapeutic Targeting, and Combinatorial Strategies

NKT cells are a specialized T cell subset that recognize lipid antigens presented by CD1d and bridge innate and adaptive immunity. Among them, iNKT cells express a semi-invariant TCR (Vα14-Jα18 in mice; Vα24-Jα18 in humans) and rapidly respond to glycolipids such as α-GalCer, whereas Type II NKT cells display broader TCR diversity and often exert immunoregulatory or suppressive functions [60,61,62,63,64]. NKT-like cells, which are not CD1d-restricted, contribute to tumor surveillance through alternative pathways [65]. The balance between iNKT and Type II NKT subsets is critical, as iNKT cells generally promote IFN-γ-dependent antitumor immunity, while Type II NKT cells can reinforce immunosuppression within the tumor microenvironment (TME) [66]. These divergent roles are shaped by transcriptional programs involving PLZF, T-bet, and RORγt [67]. Upon activation, iNKT cells rapidly secrete both Th1 (IFN-γ, IL-2, GM-CSF) and Th2 (IL-4, IL-10, IL-13) cytokines, functioning as immune rheostats that orchestrate DCs, NK cells, CTLs, and B cells [68,69]. Their activation can occur through classical CD1d-dependent presentation of exogenous or endogenous lipids [70,71], as well as CD1d-independent mechanisms involving NK receptors or cytokines such as IL-12 and IL-18 [58,72]. Microbial glycolipids from organisms including *Sphingomonas paucimobilis*, *Leishmania donovani*, and *Aspergillus fumigatus* further highlight their sentinel role in sensing infection and malignant stress [63,64].

Clinically, the protective role of iNKT cells is supported by increased tumor susceptibility in iNKT-deficient mice (e.g., Jα281^−^/^−^) [67,73] and by reduced frequency and impaired function of circulating Vα24^+^Vβ11^+^ iNKT cells in multiple human cancers, including prostate, colorectal, melanoma, breast cancer, multiple myeloma, and lung cancer [58,74,75,76,77,78,79]. Importantly, functional competence rather than absolute number often predicts clinical benefit, as demonstrated in trials where stronger IFN-γ-producing iNKT responses correlated with improved survival in non-small-cell lung cancer (NSCLC) patients (Table 1) [80]. Strategies such as α-GalCer-pulsed DCs have shown safety and the capacity to expand iNKT cells in patients [81,82,83,84], although variability in baseline iNKT levels influences outcomes [85].

α-GalCer remains the prototypical ligand for iNKT activation, inducing cytokine production and downstream NK and CTL activation [86,87]. However, repeated stimulation can trigger iNKT cell anergy and mixed Th1/Th2 polarization, limiting durable clinical efficacy [88]. This has driven the development of next-generation ligands, including Th1-biasing C20:2 analogs [89], degradation-resistant α-C-GalCer [90], and alternative small-molecule CD1d ligands designed to enhance persistence and reduce exhaustion. Combination strategies with checkpoint inhibitors (e.g., anti-PD-1) can restore IFN-γ production and counteract TME-induced dysfunction [80], whereas cytokine support (e.g., IL-12) enhances therapeutic potency in preclinical models (Table 1) [91].

Integration of iNKT ligands with chemotherapy represents a promising synergistic approach (Table 1). Chemotherapeutics such as doxorubicin and oxaliplatin induce immunogenic cell death and upregulate CD1d and NK-activating ligands, enhancing iNKT recognition [92]. Agents like cyclophosphamide and gemcitabine reduce Tregs and MDSCs, reversing tumor-induced immunosuppression [93]. Preclinical studies demonstrate improved tumor control with combinations such as paclitaxel plus α-GalCer in liposomal formulations (Table 1) [94], glycosphingolipid–doxorubicin nanocarriers [15], and cisplatin plus α-GalCer in CD1d-dependent models [95]. Low-dose chemotherapy further sensitizes tumors by upregulating death receptors (TRAIL-R2/DR5, Fas) and NK-activating ligands, enhancing immune-mediated killing [96,97,98].

Collectively, these findings position iNKT cells as central regulators of tumor immunity whose therapeutic efficacy depends on subset balance, ligand design, metabolic context, and rational combination strategies. Optimizing delivery systems, integrating immune profiling, and combining with checkpoint blockade or adoptive iNKT-based therapies will be critical to fully harness their potential in next-generation cancer immunotherapy.

**Table 1:** Preclinical and Clinical Studies on iNKT Cells in Cancer Immunotherapy.

Study Type	Intervention	Key Findings	References
Preclinical studies	Adoptive transfer of iNKT cells	iNKT cells induced strong antitumor activity in B-cell lymphoma models via IFN-γ-mediated immune responses. Type II NKT cells suppressed this activity.	[99]
	IL-12-activated iNKT cells	IL-12-activated iNKT cells inhibited metastasis and enhanced cytokine production in murine models, especially when combined with IL-18.	[72]
	CD62L+ iNKT Cells	CD62L+ iNKT cells demonstrated prolonged persistence and superior antitumor efficacy in murine leukemia models.	[22]
	CAR-iNKT Cells	CAR-iNKT cells targeting GD2 demonstrated potent antitumor activity against neuroblastoma, overcoming TME-mediated immunosuppression. Enhanced survival was noted in preclinical models of solid tumors.	[87]
	Combination with Checkpoint Inhibitors	Anti-PD-1 enhanced iNKT-mediated immunity by preventing exhaustion and improving cytokine secretion in tumor models. Combination showed synergistic effects in reducing tumor burden and improving survival.	[100]
Nanoparticle-Based Therapies	Liposomal α-GalCer	Liposomal formulations of α-GalCer reduced the risk of iNKT cell anergy, sustained IFN-γ production, and enhanced antitumor immunity in murine melanoma and lung carcinoma models.	[17]
	Glycosphingolipid-Loaded Nanoparticles	Glycosphingosomes (glycosphingolipid-loaded liposomes) significantly enhanced NKT-mediated antitumor activity in DMBA-induced tumors in mice.	[15]
	Stearylated α-GalCer Liposomes	Stearylated octaarginine-modified liposomes enhanced α-GalCer presentation, increased iNKT cell activation, and showed potent antitumor effects in highly aggressive B16 melanoma models.	[18]
	Glycolipid Analogs	Modified glycolipids targeting iNKT cells exhibited enhanced stability and Th1-biased immune responses, resulting in improved efficacy against tumors.	[91]
Phase I Clinical Trials	α-GalCer Therapy	Patients with higher baseline iNKT cell numbers demonstrated better responses to α-GalCer therapy in solid tumor trials.	[101]
	α-GalCer-Pulsed DCs	α-GalCer-loaded DCs enhanced iNKT activation, increased IFN-γ secretion, and showed efficacy in inhibiting metastasis in preclinical models. Phase I studies showed immune activation and safety in NSCLC patients.	[102]
	Monocyte-Derived DCs	α-GalCer-pulsed monocyte-derived DCs induced a 100-fold expansion of circulating iNKT cells and sustained activation in cancer patients.	[103]
	Combination Therapies	α-GalCer-pulsed PBMCs combined with IL-2/GM-CSF therapy increased survival rates in lung cancer patients with IFN-γ-producing iNKT cells.	[103]
	Allogeneic iNKT Cells	Allogeneic iNKT cell transfer reduced GvHD risk and activated host immune responses effectively. Early-phase trials in hematologic malignancies are ongoing.	[104]
Recent Innovations	CAR-iNKT Cell Engineering	CAR-iNKT cells targeting HER2 showed enhanced efficacy against HER2-positive breast cancer models. Early trials are underway.	[105]
	NK and T Cell Activation	iNKT activation induced by α-GalCer increased NK and CTL-mediated killing in various tumor models, highlighting its dual activation role.	[106]
	Dual-CAR Constructs	Dual-targeting CAR-iNKT cells (e.g., targeting GD2 and B7-H3) have been developed to address tumor antigen heterogeneity, improving tumor cell targeting and reducing the risk of immune escape.	[17]
	CRISPR-Cas9 Engineered iNKT Cells	CRISPR-Cas9 has been employed to engineer iNKT cells resistant to TME-induced suppression. These iNKT cells are modified to resist TGF-β signaling, maintaining their function in highly suppressive environments.	[99]
	iNKT Cells Expressing Cytokines	Genetically modified iNKT cells engineered to secrete IL-12 demonstrated improved cytotoxicity and TME modulation. IL-12-producing iNKT cells activated resident NK and CD8+ T cells, enhancing tumor control.	[107]
	iNKT Cells and Bispecific Antibodies	Bispecific T-cell engagers (BiTEs) targeting iNKT cells and tumor antigens have been developed to enhance iNKT cytotoxicity in solid tumors. This approach bridges iNKT cells to tumor cells, increasing immune synapse formation and tumor killing.	[108]

## 6. CAR-iNKT Cells: Engineering Precision Immunity for Cancer Therapy

**Figure 5:**
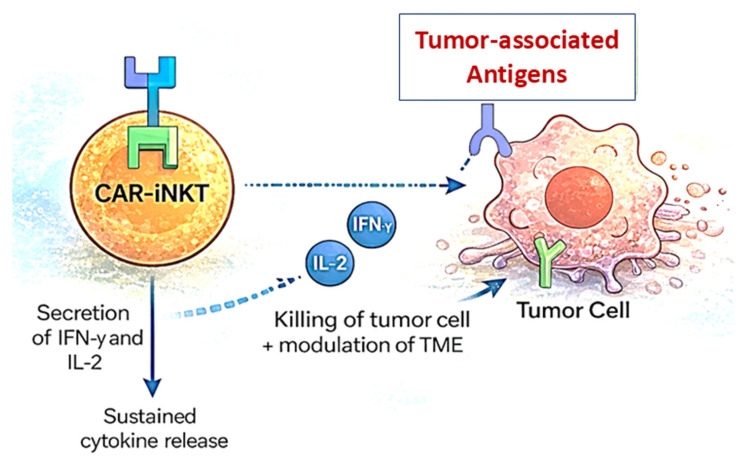
Mechanism of CAR-iNKT cells in tumor targeting. CAR-iNKT cells recognize tumor-associated antigens, such as HER2 or GD2, via their synthetic CARs, triggering the release of cytokines like IFN-γ and IL-2 and initiating potent cytotoxic responses. These cytokines not only promote direct tumor cell lysis but also reprogram TME to support broader immune activation. Compared to conventional CAR-T cells, CAR-iNKT cells demonstrate enhanced persistence and sustained cytokine secretion, offering improved therapeutic potential.

Chimeric antigen receptor (CAR)-engineered iNKT cells integrate antigen-specific targeting with the intrinsic immunoregulatory and cytotoxic properties of iNKT cells, creating a versatile and potentially safer alternative to conventional CAR-T therapy. By equipping iNKT cells with CARs directed against tumor-associated antigens, these cells gain MHC-independent, high-affinity tumor recognition while retaining CD1d restriction, thereby minimizing the risk of graft-versus-host disease in allogeneic settings and supporting off-the-shelf therapeutic strategies [109,110]. Importantly, unlike conventional T cells, iNKT cells maintain functional activity in hypoxic and immunosuppressive TMEs, a key limitation of many adoptive cell therapies. CAR-iNKT cells exert dual antitumor activity. First, they directly lyse tumor cells through CAR-mediated recognition. Second, they preserve innate iNKT effector functions, including rapid secretion of IFN-γ and TNF-α, which amplify NK cell and CTL responses and enhance dendritic cell activation (Figure 5) [111]. Simultaneously, CAR-iNKT cells suppress immunosuppressive populations such as Tregs and MDSCs, thereby remodeling the TME and overcoming key barriers to durable immunity.

Preclinical studies provide strong proof of concept. CD19-specific CAR-iNKT cells demonstrated potent cytolytic activity against B-cell leukemias and lymphomas in murine models, with effective tumor clearance and sustained persistence, in some cases outperforming conventional CAR-T cells in hostile microenvironments [83,112,113]. These findings highlight not only their antitumor efficacy but also their capacity for long-term functional stability. Clinical translation has yielded encouraging results. In a first-in-human Phase I trial, GD2-specific CAR-iNKT cells were administered to patients with relapsed or refractory neuroblastoma (Table 1). The therapy was well-tolerated, with no evidence of cytokine release syndrome (CRS) or neurotoxicity, and partial tumor regression was observed in some patients, despite advanced disease [109]. These outcomes underscore the favorable safety profile and therapeutic promise of CAR-iNKT approaches. Building on these findings, ongoing clinical trials are evaluating CAR-iNKT cells targeting HER2, CD19, CD22, and other tumor-associated antigens across hematologic and solid malignancies [113]. Current efforts are also optimizing dosing strategies, delivery routes, and combination regimens with checkpoint inhibitors or cytokine support to further enhance persistence and antitumor potency. Collectively, CAR-iNKT cells represent a next-generation cellular immunotherapy platform capable of precise tumor targeting, immune microenvironment reprogramming, and improved safety compared with conventional CAR-T strategies (Figure 5).

## 7. Enhancing iNKT Cell-Based Cancer Therapy Through Nano-Formulations and CAR Engineering

**Figure 6:**
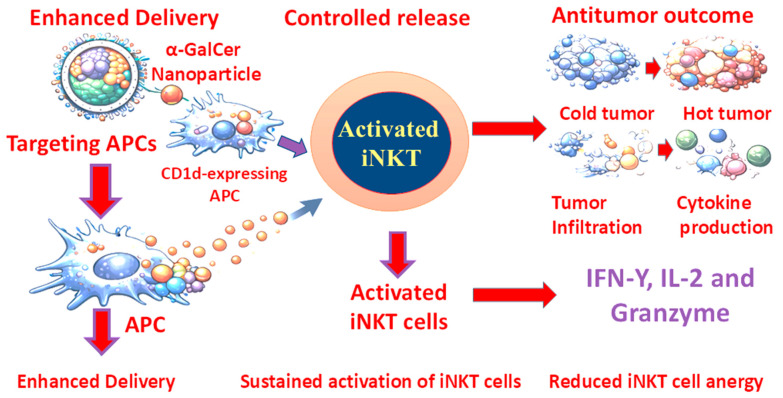
Nanoparticle-Mediated α-GalCer Delivery Enhances iNKT Cell Antitumor Immunity Without Inducing Anergy. Lipid-engineered nanoparticles improve α-GalCer stability and selectively target CD1d-expressing APCs. Following uptake, controlled release enables sustained CD1d presentation to iNKT cells, resulting in the activation without inducing anergy. Activated iNKT cells produce IFN-γ, IL-2, and granzyme, promoting tumor infiltration, cytokine production, and conversion of “cold” tumors into “hot” immunologically active sites, thereby enhancing antitumor immunity.

Although α-GalCer and its structural analogs have demonstrated preclinical efficacy in activating iNKT cells and initiating antitumor immunity, their clinical translation remains limited by rapid systemic clearance, poor bioavailability, suboptimal tumor targeting, and the induction of iNKT cell anergy upon repeated stimulation. These limitations reduce both the magnitude and durability of therapeutic responses. To address these challenges, lipid-engineered nano-formulation strategies have emerged as transformative platforms capable of optimizing pharmacokinetics, improving antigen presentation, and reshaping immune activation dynamics (Figure 6) [12,15].

### 7.1. Nanoparticle Engineering to Improve Stability, Bioavailability, and Targeted Delivery

One of the principal advantages of nanoparticle-based systems lies in their ability to enhance the stability and bioavailability of α-GalCer [12]. Encapsulation within liposomes, polymeric nanoparticles, micelles, or lipid-based nanocarriers shields α-GalCer from premature enzymatic degradation and rapid hepatic clearance, thereby prolonging systemic circulation [114]. This protective effect enhances the likelihood of delivery to lymphoid tissues and tumor-draining lymph nodes, where iNKT priming predominantly occurs. Importantly, nanoparticle formulations enable preferential targeting of CD1d-expressing APCs, particularly DCs [114]. By facilitating APC-restricted uptake, nano-formulations improve CD1d-mediated glycolipid presentation while minimizing non-professional antigen presentation, a key contributor to iNKT anergy. Liposomal α-GalCer formulations have demonstrated superior DC uptake and more sustained iNKT activation as compared to free ligand administration. Similarly, glycosphingolipid-loaded liposomes derived from *Sphingomonas paucimobilis* enhanced antitumor immunity through improved ligand presentation efficiency [17]. Surface functionalization strategies, including glycan-coated nanoparticles or ligand-decorated carriers, further refine targeting specificity toward CD1d^+^ APC subsets [114]. These approaches enhance spatial precision, reduce systemic cytokine bursts, and promote tumor-focused immune activation.

### 7.2. Controlled Release Kinetics: Preventing Anergy and Promoting Th1 Polarization

Repeated systemic administration of soluble α-GalCer frequently induces iNKT cell hyporesponsiveness (anergy), limiting long-term efficacy [12]. Nano-formulations address this issue through controlled and sustained release kinetics, ensuring gradual antigen exposure rather than acute overstimulation (Figure 6). Stearylated α-GalCer incorporated into lipid nanoparticles has been shown to induce prolonged cytokine production while preserving iNKT responsiveness in tumor-bearing mice [12]. By avoiding excessive early activation, nanoparticle-mediated delivery promotes sustained Th1-biased responses, characterized by enhanced IFN-γ production and durable activation of NK cells and CTLs. This controlled immunological tuning enhances antitumor immunity while mitigating functional exhaustion. In murine melanoma models, polymeric nanoparticle-encapsulated α-GalCer significantly elevated IFN-γ levels and improved tumor regression compared with soluble ligand [114], underscoring the importance of pharmacodynamic modulation in optimizing immune outcomes.

### 7.3. Co-Delivery Strategies: Synergistic Immune Amplification

Nanoparticles also serve as multifunctional platforms enabling co-delivery of iNKT ligands with additional immunomodulatory agents, thereby amplifying immune synergy. Co-encapsulation of α-GalCer with toll-like receptor (TLR) agonists enhances DC maturation, co-stimulatory molecule expression, and IL-12 production, collectively strengthening iNKT activation and downstream CTL priming [115,116,117,118]. These dual-delivery systems generate strong pro-inflammatory cascade within the TME. Similarly, nanoparticles can be engineered to deliver α-GalCer alongside cytokines (e.g., IL-12, IL-18) or chemotherapeutic agents that induce immunogenic cell death. Such combinations enhance antigen availability, increase CD1d expression, and promote cross-priming of tumor-specific T cells. This strategy transforms nano-formulations from simple delivery vehicles into integrated immuno-engineering platforms capable of orchestrating multi-layered immune activation.

### 7.4. Reprogramming the Tumor Microenvironment: Converting “Cold” Tumors into “Hot” Tumors

A major barrier in cancer immunotherapy is the presence of immunologically “cold” tumors characterized by low effector T-cell infiltration and high suppressive cell populations. Nanoparticle-mediated iNKT activation has demonstrated the capacity to reshape the TME into an immune-active state. By enhancing IFN-γ and TNF-α production, nano-delivered α-GalCer promotes NK and CTL recruitment DC activation, and suppression of regulatory cell subsets. In melanoma-bearing mice, lipid nanoparticle delivery of α-GalCer markedly suppressed tumor progression and increased effector immune infiltration [114]. Multifunctional nanoparticle systems further potentiate these effects by amplifying inflammatory signaling within the tumor bed. Through coordinated cytokine release and APC licensing, nanoparticle platforms can effectively convert immune-excluded tumors into inflamed, therapeutically responsive environments (Figure 6).

### 7.5. Translational Challenges and Future Considerations

Despite promising preclinical outcomes, several translational challenges must be addressed before nanoparticle-enhanced iNKT therapies can be broadly implemented in the clinic. A primary concern is scalable and reproducible manufacturing. Strict control of nanoparticle size, surface properties, ligand loading, and release kinetics is essential to ensure consistent pharmacokinetics and immune responses. Variability in formulation parameters may significantly affect biodistribution and therapeutic performance. Optimizing tissue targeting also remains critical to maximize delivery to lymphoid organs or tumor sites while limiting non-specific accumulation. In addition, long-term physicochemical stability under clinical manufacturing, storage, and transport conditions must be validated to maintain product integrity. Finally, regulatory approval of complex nanomedicine platforms requires rigorous evaluation of quality control, reproducibility, and compliance with established safety standards. Addressing these issues will require coordinated interdisciplinary efforts spanning immunology, materials science, pharmacology, and regulatory science to facilitate successful clinical translation.

### 7.6. Therapeutic Outlook

Collectively, lipid-engineered nanoparticle systems represent a next-generation strategy to enhance iNKT cell-mediated cancer immunotherapy. By improving α-GalCer stability, enabling APC-targeted delivery, preventing anergy through controlled release, facilitating synergistic co-delivery approaches, and reshaping the tumor microenvironment, these platforms substantially expand the therapeutic potential of iNKT-based strategies. Continued refinement and clinical translation of these nano-engineering approaches may establish durable, tumor-specific immune activation as a central pillar of future cancer immunotherapy.

## 8. Safety, Toxicity, and Translational Considerations of CAR-iNKT and Nanoparticle-Based Therapies

Although CAR-iNKT cells and lipid-engineered nanoparticle platforms are highlighted as potentially safer alternatives to conventional CAR-T and systemic immunotherapies, important safety considerations must be carefully addressed to ensure successful clinical translation. CAR-iNKT cells have demonstrated a favorable safety profile in early-phase clinical studies, with low incidence of cytokine release syndrome and neurotoxicity as compared to conventional CAR-T therapies [119,120]. Nevertheless, given the rapid production of Th1 cytokines such as IFN-γ and TNF-α, there remains a theoretical risk of systemic inflammatory responses. Careful dose optimization, stepwise infusion strategies, and close immune monitoring are therefore essential in future trials.

Because iNKT cells recognize CD1d-expressing cells and orchestrate broad immune activation, unintended targeting of non-malignant tissues or excessive activation of bystander immune populations may occur. Strategies such as tumor-restricted CAR targeting, controlled cytokine expression, and precision nanoparticle delivery to professional APCs are critical to minimize off-target immune effects. Lipid-engineered nanoparticles improve pharmacokinetics and APC targeting; however, biodistribution must be tightly controlled. Non-specific hepatic accumulation, systemic dispersion, or excessive immune stimulation may compromise safety. As discussed in the translational challenges section, reproducible manufacturing, strict control of particle size and surface characteristics, and thorough evaluation of immunogenicity are essential to reduce toxicity risks. Long-term stability, storage conditions, and batch-to-batch consistency must also be validated to ensure predictable clinical outcomes.

For both genetically engineered CAR-iNKT cells and nanoparticle-based ligand delivery, long-term safety data remain limited. Extended follow-up is required to assess durability of response, immune memory formation, delayed toxicities, and potential immune dysregulation after repeated administration. Collectively, while current evidence supports a comparatively favorable safety profile, comprehensive preclinical toxicology studies, standardized manufacturing protocols, and carefully designed clinical trials are indispensable to fully establish the safety and long-term clinical viability of these emerging immunotherapeutic strategies.

## 9. Future Directions

Advances in iNKT cell-based cancer immunotherapy, particularly through lipid-engineered nanoparticle delivery systems and CAR-iNKT cell engineering, provide a strong foundation for next-generation therapeutic strategies. To maximize clinical benefit, continued optimization of nanoparticle platforms is essential. Improving ligand stability, pharmacokinetics, and selective delivery to professional antigen-presenting cells will enhance sustained iNKT activation while minimizing systemic exposure. Surface functionalization approaches and biodegradable carrier systems may further refine tissue targeting and immune precision. In addition, incorporating complementary immunostimulatory molecules into nano-formulations could strengthen antitumor immune priming within suppressive tumor environments.

Simultaneously, refinement of CAR-iNKT cell engineering is required to improve tumor specificity, persistence, and functional stability. Gene-editing technologies offer opportunities to enhance resistance to inhibitory signals within the tumor microenvironment and to optimize effector programming. Rational CAR design, including improved signaling domains, may further augment in vivo expansion and cytotoxic capacity while maintaining safety. Therapeutic efficacy is also likely to benefit from rational combination strategies. Coordinated integration with immune checkpoint blockade, cytokine support, or other cellular therapies may enhance immune amplification and durability of response. A deeper understanding of how iNKT cells interact with dendritic cells, NK cells, and conventional T cells will be critical for designing synergistic regimens that overcome tumor-driven immune suppression.

Given inter-patient variability in iNKT cell frequency and function, immune profiling may enable more precise patient selection and individualized therapeutic planning. Development of standardized, scalable manufacturing protocols for both engineered cells and nano-formulations will be indispensable for regulatory approval and widespread clinical implementation. Beyond oncology, the immunomodulatory properties of iNKT cells may support broader applications in other immune-mediated diseases, further expanding their translational potential.

## 10. Conclusions

iNKT cells represent a unique and powerful immunotherapeutic platform at the intersection of innate and adaptive immunity. Their rapid cytokine secretion, ability to activate dendritic cells, NK cells, and cytotoxic T lymphocytes, and capacity to reshape the tumor microenvironment (TME) position them as central orchestrators of antitumor immunity. In cancers characterized by immune exhaustion and chemotherapy-induced immunosuppression, iNKT cells offer a means to restore immune competence and reinitiate coordinated antitumor responses. However, clinical translation of iNKT-based therapies has been historically limited by several challenges, most notably the induction of anergy following repeated α-GalCer administration and suboptimal pharmacokinetics of soluble ligands. The emergence of lipid-engineered nanoparticle platforms provides a transformative solution to these limitations. By enhancing ligand stability, enabling APC-targeted delivery, and allowing controlled antigen release, nanotechnology mitigates overstimulation-induced anergy while promoting sustained Th1-biased activation. Beyond serving as delivery vehicles, multifunctional nanoparticles can co-deliver immunomodulators or chemotherapeutics, thereby converting immunologically “cold” tumors into inflamed, responsive microenvironments. In parallel, CAR-iNKT cell engineering introduces antigen-specific precision to the intrinsic immunoregulatory capacity of iNKT cells. CAR modification enables MHC-independent tumor targeting while preserving CD1d-restricted functions, allowing dual mechanisms of tumor eradication: direct cytotoxicity and immune orchestration. Importantly, early clinical data suggest a favorable safety profile compared to conventional CAR-T therapies, highlighting their translational promise. The integration of gene-editing technologies and optimized CAR constructs further expands the therapeutic potential of this platform.

Rational combination strategies further amplify these advances. Chemotherapy can enhance tumor immunogenicity and reduce suppressive immune subsets, thereby sensitizing tumors to iNKT-mediated killing. Similarly, immune checkpoint blockade and cytokine support can restore effector function and overcome TME-induced inhibition. Together, these multimodal approaches create a synergistic framework in which nano-delivery systems, cellular engineering, and combination immunotherapy converge to produce durable and amplified antitumor immunity. While non-invariant CD1d-restricted NKT cells may exert immunosuppressive Th2-skewed effects, IFN-γ-producing iNKT cells are consistently associated with improved cancer outcomes. Although manipulation of non-invariant NKT subsets remains challenging due to limited mechanistic understanding, the relative rarity of iNKT cells presents a therapeutic advantage: they can be safely expanded or engineered without disrupting broader T cell homeostasis.

Collectively, the convergence of nanotechnology, CAR engineering, and combinatorial immunomodulation establishes iNKT cell-based therapy as a next-generation immunotherapeutic paradigm. Continued refinement of delivery platforms, genetic engineering strategies, and translational protocols will be essential to fully harness their potential. With these advances, iNKT-targeted approaches may evolve from experimental interventions into clinically robust strategies capable of overcoming tumor immune evasion and improving outcomes in resistant and immunosuppressive cancers.

## Data Availability

No new data were created or analyzed in this study. Data sharing is not applicable to this article.

## References

[1] Ferlay J., Colombet M., Soerjomataram I., Mathers C., Parkin D.M., Piñeros M., Znaor A., Bray F. (2019). Estimating the global cancer incidence and mortality in 2018: GLOBOCAN sources and methods. Int. J. Cancer.

[2] Leslie P.L., Chao Y.L., Tsai Y.H., Ghosh S.K., Porrello A., Van Swearingen A.E.D., Harrison E.B., Cooley B.C., Parker J.S., Carey L.A. (2019). Histone deacetylase 11 inhibition promotes breast cancer metastasis from lymph nodes. Nat. Commun..

[3] Juthani R., Punatar S., Mittra I. (2024). New light on chemotherapy toxicity and its prevention. BJC Rep..

[4] Arafat Hossain M. (2024). A comprehensive review of immune checkpoint inhibitors for cancer treatment. Int. Immunopharmacol..

[5] Posa M.K., Singh J., Parveen S., Panda S. (2026). CAR T-cell therapy: A promising novel approach for treatment of cancer. Cancer Treat. Res. Commun..

[6] Bendelac A., Savage P.B., Teyton L. (2007). The biology of NKT cells. Annu. Rev. Immunol..

[7] Brutkiewicz R.R., Lin Y., Cho S., Hwang Y.K., Sriram V., Roberts T.J. (2003). CD1d-mediated antigen presentation to natural killer T (NKT) cells. Crit. Rev. Immunol..

[8] Brutkiewicz R.R., Sriram V. (2002). Natural killer T (NKT) cells and their role in antitumor immunity. Crit. Rev. Oncol. Hematol..

[9] King L.A., Lameris R., de Gruijl T.D., van der Vliet H.J. (2018). CD1d-Invariant Natural Killer T Cell-Based Cancer Immunotherapy: α-Galactosylceramide and Beyond. Front. Immunol..

[10] Sullivan B.A., Kronenberg M. (2005). Activation or anergy: NKT cells are stunned by alpha-galactosylceramide. J. Clin. Investig..

[11] Park Y.H., Lee S.W., Kim T.C., Park H.J., Van Kaer L., Hong S. (2024). The iNKT cell ligand α-GalCer prevents murine septic shock by inducing IL10-producing iNKT and B cells. Front. Immunol..

[12] Thapa P., Zhang G., Xia C., Gelbard A., Overwijk W.W., Liu C., Hwu P., Chang D.Z., Courtney A., Sastry J.K. (2009). Nanoparticle formulated alpha-galactosylceramide activates NKT cells without inducing anergy. Vaccine.

[13] Motohashi S., Okamoto Y., Yoshino I., Nakayama T. (2011). Anti-tumor immune responses induced by iNKT cell-based immunotherapy for lung cancer and head and neck cancer. Clin. Immunol..

[14] Ghinnagow R., De Meester J., Cruz L.J., Aspord C., Corgnac S., Macho-Fernandez E., Soulard D., Fontaine J., Chaperot L., Charles J. (2017). Co-delivery of the NKT agonist α-galactosylceramide and tumor antigens to cross-priming dendritic cells breaks tolerance to self-antigens and promotes antitumor responses. Oncoimmunology.

[15] Khan M.A., Aljarbou A.N., Aldebasi Y.H., Alorainy M.S., Rahmani A.H., Younus H., Khan A. (2018). Liposomal formulation of glycosphingolipids from Sphingomonas paucimobilis induces antitumour immunity in mice. J. Drug Target..

[16] Nakamura T., Yamazaki D., Yamauchi J., Harashima H. (2013). The nanoparticulation by octaarginine-modified liposome improves α-galactosylceramide-mediated antitumor therapy via systemic administration. J. Control. Release.

[17] Wang Z., Zhang G. (2024). CAR-iNKT cell therapy: Mechanisms, advantages, and challenges. Curr. Res. Transl. Med..

[18] Wang Y., Bhave M.S., Yagita H., Cardell S.L. (2020). Natural Killer T-Cell Agonist α-Galactosylceramide and PD-1 Blockade Synergize to Reduce Tumor Development in a Preclinical Model of Colon Cancer. Front. Immunol..

[19] Hammond T.C., Purbhoo M.A., Kadel S., Ritz J., Nikiforow S., Daley H., Shaw K., van Besien K., Gomez-Arteaga A., Stevens D. (2024). A phase 1/2 clinical trial of invariant natural killer T cell therapy in moderate-severe acute respiratory distress syndrome. Nat. Commun..

[20] Adams J.L., Smothers J., Srinivasan R., Hoos A. (2015). Big opportunities for small molecules in immuno-oncology. Nat. Rev. Drug Discov..

[21] Spranger S., Sivan A., Corrales L., Gajewski T.F. (2016). Tumor and Host Factors Controlling Antitumor Immunity and Efficacy of Cancer Immunotherapy. Adv. Immunol..

[22] Pandya P.H., Murray M.E., Pollok K.E., Renbarger J.L. (2016). The Immune System in Cancer Pathogenesis: Potential Therapeutic Approaches. J. Immunol. Res..

[23] Vesely M.D., Kershaw M.H., Schreiber R.D., Smyth M.J. (2011). Natural innate and adaptive immunity to cancer. Annu. Rev. Immunol..

[24] Sims G.P., Rowe D.C., Rietdijk S.T., Herbst R., Coyle A.J. (2010). HMGB1 and RAGE in inflammation and cancer. Annu. Rev. Immunol..

[25] Schreiber R.D., Old L.J., Smyth M.J. (2011). Cancer immunoediting: Integrating immunity’s roles in cancer suppression and promotion. Science.

[26] Mittal D., Gubin M.M., Schreiber R.D., Smyth M.J. (2014). New insights into cancer immunoediting and its three component phases—Elimination, equilibrium and escape. Curr. Opin. Immunol..

[27] Calì B., Molon B., Viola A. (2017). Tuning cancer fate: The unremitting role of host immunity. Open Biol..

[28] Aguirre-Ghiso J.A. (2007). Models, mechanisms and clinical evidence for cancer dormancy. Nat. Rev. Cancer.

[29] Koebel C.M., Vermi W., Swann J.B., Zerafa N., Rodig S.J., Old L.J., Smyth M.J., Schreiber R.D. (2007). Adaptive immunity maintains occult cancer in an equilibrium state. Nature.

[30] Teng M.W., Vesely M.D., Duret H., McLaughlin N., Towne J.E., Schreiber R.D., Smyth M.J. (2012). Opposing roles for IL-23 and IL-12 in maintaining occult cancer in an equilibrium state. Cancer Res..

[31] Wang H.F., Wang S.S., Huang M.C., Liang X.H., Tang Y.J., Tang Y.L. (2019). Targeting Immune-Mediated Dormancy: A Promising Treatment of Cancer. Front. Oncol..

[32] Montagna D., Maccario R., Locatelli F., Montini E., Pagani S., Bonetti F., Daudt L., Turin I., Lisini D., Garavaglia C. (2006). Emergence of antitumor cytolytic T cells is associated with maintenance of hematologic remission in children with acute myeloid leukemia. Blood.

[33] Angell T.E., Lechner M.G., Jang J.K., LoPresti J.S., Epstein A.L. (2014). MHC class I loss is a frequent mechanism of immune escape in papillary thyroid cancer that is reversed by interferon and selumetinib treatment in vitro. Clin. Cancer Res..

[34] Jones L.M., Broz M.L., Ranger J.J., Ozcelik J., Ahn R., Zuo D., Ursini-Siegel J., Hallett M.T., Krummel M., Muller W.J. (2016). STAT3 Establishes an Immunosuppressive Microenvironment during the Early Stages of Breast Carcinogenesis to Promote Tumor Growth and Metastasis. Cancer Res..

[35] Tian M., Zhang Y., Liu Z., Sun G., Mor G., Liao A. (2016). The PD-1/PD-L1 inhibitory pathway is altered in pre-eclampsia and regulates T cell responses in pre-eclamptic rats. Sci. Rep..

[36] Brutkiewicz R.R., Yunes-Medina L., Liu J. (2018). Immune evasion of the CD1d/NKT cell axis. Curr. Opin. Immunol..

[37] Setiadi A.F., Omilusik K., David M.D., Seipp R.P., Hartikainen J., Gopaul R., Choi K.B., Jefferies W.A. (2008). Epigenetic enhancement of antigen processing and presentation promotes immune recognition of tumors. Cancer Res..

[38] Shastri N., Nagarajan N., Lind K.C., Kanaseki T. (2014). Monitoring peptide processing for MHC class I molecules in the endoplasmic reticulum. Curr. Opin. Immunol..

[39] van der Burg S.H., Arens R., Ossendorp F., van Hall T., Melief C.J. (2016). Vaccines for established cancer: Overcoming the challenges posed by immune evasion. Nat. Rev. Cancer.

[40] Muenst S., Läubli H., Soysal S.D., Zippelius A., Tzankov A., Hoeller S. (2016). The immune system and cancer evasion strategies: Therapeutic concepts. J. Intern. Med..

[41] Bailey J.C., Iyer A.K., Renukaradhya G.J., Lin Y., Nguyen H., Brutkiewicz R.R. (2014). Inhibition of CD1d-mediated antigen presentation by the transforming growth factor-β/Smad signalling pathway. Immunology.

[42] Hix L.M., Shi Y.H., Brutkiewicz R.R., Stein P.L., Wang C.R., Zhang M. (2011). CD1d-expressing breast cancer cells modulate NKT cell-mediated antitumor immunity in a murine model of breast cancer metastasis. PLoS ONE.

[43] Alizadeh D., Larmonier N. (2014). Chemotherapeutic targeting of cancer-induced immunosuppressive cells. Cancer Res..

[44] Apetoh L., Végran F., Ladoire S., Ghiringhelli F. (2011). Restoration of antitumor immunity through selective inhibition of myeloid-derived suppressor cells by anticancer therapies. Curr. Mol. Med..

[45] De Santo C., Salio M., Masri S.H., Lee L.Y., Dong T., Speak A.O., Porubsky S., Booth S., Veerapen N., Besra G.S. (2008). Invariant NKT cells reduce the immunosuppressive activity of influenza A virus-induced myeloid-derived suppressor cells in mice and humans. J. Clin. Investig..

[46] Yoneda K., Morii T., Nieda M., Tsukaguchi N., Amano I., Tanaka H., Yagi H., Narita N., Kimura H. (2005). The peripheral blood Vα24^+^ NKT cell numbers decrease in patients with haematopoietic malignancy. Leuk. Res..

[47] Molling J.W., Kölgen W., van der Vliet H.J., Boomsma M.F., Kruizenga H., Smorenburg C.H., Molenkamp B.G., Langendijk J.A., Leemans C.R., von Blomberg B.M. (2005). Peripheral blood IFN-γ-secreting Vα24^+^Vβ11^+^ NKT cell numbers are decreased in cancer patients independent of tumor type or tumor load. Int. J. Cancer.

[48] Fujii S., Shimizu K., Klimek V., Geller M.D., Nimer S.D., Dhodapkar M.V. (2003). Severe and selective deficiency of interferon-γ-producing invariant natural killer T cells in patients with Myelodysplastic syndromes. Br. J. Haematol..

[49] Motohashi S., Nakayama T. (2008). Clinical applications of natural killer T cell-based immunotherapy for cancer. Cancer Sci..

[50] Quail D.F., Joyce J.A. (2013). Microenvironmental regulation of tumor progression and metastasis. Nat. Med..

[51] Qin X., Li T., Li S., Yang H., Wu C., Zheng C., You F., Liu Y. (2020). The tumor biochemical and biophysical microenvironments synergistically contribute to cancer cell malignancy. Cell. Mol. Immunol..

[52] Wang Y., Li Y.R. (2024). Harnessing Chimeric Antigen Receptor-engineered Invariant Natural Killer T Cells: Therapeutic Strategies for Cancer and the Tumor Microenvironment. Curr. Pharm. Biotechnol..

[53] Arner E.N., Rathmell J.C. (2023). Metabolic programming and immune suppression in the tumor microenvironment. Cancer Cell.

[54] Lindau D., Gielen P., Kroesen M., Wesseling P., Adema G.J. (2013). The immunosuppressive tumour network: Myeloid-derived suppressor cells, regulatory T cells and natural killer T cells. Immunology.

[55] Tognarelli E.I., Gutiérrez-Vera C., Palacios P.A., Pasten-Ferrada I.A., Aguirre-Muñoz F., Cornejo D.A., González P.A., Carreño L.J. (2023). Natural Killer T Cell Diversity and Immunotherapy. Cancers.

[56] King I.L., Amiel E., Tighe M., Mohrs K., Veerapen N., Besra G., Mohrs M., Leadbetter E.A. (2013). The mechanism of splenic invariant NKT cell activation dictates localization in vivo. J. Immunol..

[57] Smyth M.J., Thia K.Y.T., Street S.E., Cretney E., Trapani J.A., Taniguchi M., Kawano T., Pelikan S.B., Crowe N.Y., Godfrey D.I. (2000). Differential tumor surveillance by natural killer (NK) and NKT cells. J. Exp. Med..

[58] Fujii S., Shimizu K., Kronenberg M., Steinman R.M. (2002). Prolonged IFN-γ-producing NKT response induced with α-galactosylceramide-loaded DCs. Nat. Immunol..

[59] Terabe M., Berzofsky J.A. (2014). The immunoregulatory role of type I and type II NKT cells in cancer and other diseases. Cancer Immunol. Immunother..

[60] Godfrey D.I., Stankovic S., Baxter A. (2010). Raising the NKT cell family. Nat. Immunol..

[61] Khan M.A., Khan A. (2021). Role of NKT Cells during Viral Infection and the Development of NKT Cell-Based Nanovaccines. Vaccines.

[62] Berzins S.P., Smyth M., Baxter A. (2011). Presumed guilty: Natural killer T cell defects and human disease. Nat. Rev. Immunol..

[63] Sriram V., Du W., Gervay-Hague J., Brutkiewicz R.R. (2005). Cell wall glycosphingolipids of Sphingomonas paucimobilis are CD1d-specific ligands for NKT cells. Eur. J. Immunol..

[64] Brutkiewicz R.R. (2006). CD1d ligands: The good, the bad, and the ugly. J. Immunol..

[65] Waldowska M., Bojarska-Junak A., Roliński J. (2017). A brief review of clinical trials involving manipulation of invariant NKT cells as a promising approach in future cancer therapies. Cent. Eur. J. Immunol..

[66] Renukaradhya G.J., Khan M.A., Vieira M., Du W., Gervay-Hague J., Brutkiewicz R.R. (2008). Type I NKT cells protect (and type II NKT cells suppress) the host’s innate antitumor immune response to a B-cell lymphoma. Blood.

[67] Singh A.K., Tripathi P., Cardell S.L. (2018). Type II NKT Cells: An Elusive Population With Immunoregulatory Properties. Front. Immunol..

[68] Tang X.Z., Jo J., Tan A.T., Sandalova E., Chia A., Tan K.C., Lee K.H., Gehring A., De Libero G., Bertoletti A. (2013). IL-7 Licenses Activation of Human Liver Intrasinusoidal Mucosal-Associated Invariant T Cells. J. Immunol..

[69] Loh L., Wang Z., Sant S., Koutsakos M., Jegaskanda S., Corbett A.J., Liu L., Fairlie D., Crowe J., Rossjohn J. (2016). Human mucosal-associated invariant T cells contribute to antiviral influenza immunity via IL-18-dependent activation. Proc. Natl. Acad. Sci. USA.

[70] Van Kaer L., Wu L., Joyce S. (2016). Mechanisms and Consequences of Antigen Presentation by CD1. Trends Immunol..

[71] Roberts T.J., Sriram V., Spence P.M., Gui M., Hayakawa K., Bacik I., Bennink J.R., Yewdell J.W., Brutkiewicz R.R. (2002). Recycling CD1d1 molecules present endogenous antigens processed in an endocytic compartment to NKT cells. J. Immunol..

[72] Krijgsman D., Hokland M., Kuppen P.J.K. (2018). The Role of Natural Killer T Cells in Cancer—A Phenotypical and Functional Approach. Front. Immunol..

[73] Smyth M.J., Crowe N.Y., Godfrey D.I. (2001). NK cells and NKT cells collaborate in host protection from methylcholanthrene-induced fibrosarcoma. Int. Immunol..

[74] Tahir S.M., Cheng O., Shaulov A., Koezuka Y., Bubley G.J., Wilson S.B., Balk S.P., Exley M.A. (2001). Loss of IFN-γ production by invariant NK T cells in advanced cancer. J. Immunol..

[75] McEwen-Smith R.M., Salio M., Cerundolo V. (2015). The regulatory role of invariant NKT cells in tumor immunity. Cancer Immunol. Res..

[76] Lynch L., O’Shea D., Winter D.C., Geoghegan J., Doherty D.G., O’Farrelly C. (2009). Invariant NKT cells and CD1d^+^ cells amass in human omentum and are depleted in patients with cancer and obesity. Eur. J. Immunol..

[77] Crough T., Purdie D.M., Okai M., Maksoud A., Nieda M., Nicol A.J. (2004). Modulation of human Vα24^+^Vβ11^+^ NKT cells by age, malignancy and conventional anticancer therapies. Br. J. Cancer.

[78] Tachibana T., Onodera H., Tsuruyama T., Mori A., Nagayama S., Hiai H., Imamura M. (2005). Increased intratumor Vα24-positive natural killer T cells: A prognostic factor for primary colorectal carcinomas. Clin. Cancer Res..

[79] Schneiders F.L., de Bruin R.C., van den Eertwegh A.J., Scheper R.J., Leemans C.R., Brakenhoff R.H., Langendijk J.A., Verheul H.M., de Gruijl T.D., Molling J.W. (2012). Circulating invariant natural killer T-cell numbers predict outcome in head and neck squamous cell carcinoma: Updated analysis with 10-year follow-up. J. Clin. Oncol..

[80] Giaccone G., Punt C.J., Ando Y., Ruijter R., Nishi N., Peters M., von Blomberg B.M., Scheper R.J., van der Vliet H.J., van den Eertwegh A.J. (2002). A phase I study of the natural killer T-cell ligand alpha-galactosylceramide (KRN7000) in patients with solid tumors. Clin. Cancer Res..

[81] Ishikawa A., Motohashi S., Ishikawa E., Fuchida H., Higashino K., Otsuji M., Iizasa T., Nakayama T., Taniguchi M., Fujisawa T. (2005). A phase I study of α-galactosylceramide (KRN7000)-pulsed dendritic cells in patients with advanced and recurrent non-small cell lung cancer. Clin. Cancer Res..

[82] Konishi J., Yamazaki K., Yokouchi H., Shinagawa N., Iwabuchi K., Nishimura M. (2004). The characteristics of human NKT cells in lung cancer—CD1d-independent cytotoxicity against lung cancer cells by NKT cells and decreased human NKT cell response in lung cancer patients. Hum. Immunol..

[83] Hadiloo K., Tahmasebi S., Esmaeilzadeh A. (2023). CAR-NKT cell therapy: A new promising paradigm of cancer immunotherapy. Cancer Cell Int..

[84] Chang D.H., Osman K., Connolly J., Kukreja A., Krasovsky J., Pack M., Hutchinson A., Geller M., Liu N., Annable R. (2007). Sustained expansion of NKT cells and antigen-specific T cells after injection of α-galactosylceramide-loaded mature dendritic cells in cancer patients. J. Exp. Med..

[85] Nair S., Dhodapkar M.V. (2017). Natural killer T cells in cancer immunotherapy. Front. Immunol..

[86] Kawano T., Cui J., Koezuka Y., Toura I., Kaneko Y., Motoki K., Ueno H., Nakagawa R., Sato H., Kondo E. (1997). CD1d-restricted and TCR-mediated activation of Vα14 NKT cells by glycosylceramides. Science.

[87] Coquet J.M., Chakravarti S., Kyparissoudis K., McNab F.W., Pitt L.A., McKenzie B.S., Berzins S.P., Smyth M.J., Godfrey D.I. (2008). Diverse cytokine production by NKT cell subsets and identification of an IL-17-producing CD4^−^NK1.1^−^ NKT cell population. Proc. Natl. Acad. Sci. USA.

[88] Parekh V.V., Wilson M.T., Olivares-Villagómez D., Singh A.K., Wu L., Wang C.R., Joyce S., Van Kaer L. (2005). Glycolipid antigen induces long-term natural killer T cell anergy in mice. J. Clin. Investig..

[89] Yu E.D., Girardi E., Wang J., Mac T.T., Yu K.O., Van Calenbergh S., Porcelli S.A., Zajonc D.M. (2012). Structural basis for the recognition of C20:2-αGalCer by the invariant natural killer T cell receptor-like antibody L363. J. Biol. Chem..

[90] Aspeslagh S., Nemčovič M., Pauwels N., Venken K., Wang J., Van Calenbergh S., Zajonc D.M., Elewaut D. (2013). Enhanced TCR footprint by a novel glycolipid increases NKT-dependent tumor protection. J. Immunol..

[91] Shin T., Nakayama T., Akutsu Y., Motohashi S., Shibata Y., Harada M., Kamada N., Shimizu C., Shimizu E., Saito T. (2001). Inhibition of tumor metastasis by adoptive transfer of IL-12-activated Vα14 NKT cells. Int. J. Cancer.

[92] Gebremeskel S., Lobert L., Tanner K., Walker B., Oliphant T., Clarke L.E., Dellaire G., Johnston B. (2017). Natural Killer T-cell Immunotherapy in Combination with Chemotherapy-Induced Immunogenic Cell Death Targets Metastatic Breast Cancer. Cancer Immunol. Res..

[93] Desai S.A., Patel V.P., Bhosle K.P., Nagare S.D., Thombare K.C. (2025). The tumor microenvironment: Shaping cancer progression and treatment response. J. Chemother..

[94] Yang Y., Tai X., Shi K., Ruan S., Qiu Y., Zhang Z., Xiang B., He Q. (2016). A New Concept of Enhancing Immuno-Chemotherapeutic Effects Against B16F10 Tumor via Systemic Administration by Taking Advantages of the Limitation of EPR Effect. Theranostics.

[95] Wu L., Yun Z., Tagawa T., De la Maza L., Wu M.O., Yu J., Zhao Y., de Perrot M. (2014). Activation of CD1d-restricted natural killer T cells can inhibit cancer cell proliferation during chemotherapy by promoting the immune responses in murine mesothelioma. Cancer Immunol. Immunother..

[96] Fallarini S., Paoletti T., Orsi Battaglini N., Lombardi G. (2012). Invariant NKT cells increase drug-induced osteosarcoma cell death. Br. J. Pharmacol..

[97] Mattarollo S.R., Kenna T., Nieda M., Nicol A.J. (2006). Chemotherapy pretreatment sensitizes solid tumor-derived cell lines to Vα24^+^ NKT cell-mediated cytotoxicity. Int. J. Cancer.

[98] Aketa H., Tatsumi T., Kohga K., Tsunematsu H., Aono S., Shimizu S., Kodama T., Nawa T., Shigekawa M., Hikita H. (2013). The combination therapy of α-galactosylceramide and 5-fluorouracil showed antitumor effect synergistically against liver tumor in mice. Int. J. Cancer.

[99] Rotolo A., Caputo V.S., Holubova M., Baxan N., Dubois O., Chaudhry M.S., Xiao X., Goudevenou K., Pitcher D.S., Petevi K. (2018). Enhanced anti-lymphoma activity of CAR19-iNKT cells underpinned by dual CD19 and CD1d targeting. Cancer Cell.

[100] Finn O.J. (2012). Immuno-oncology: Understanding the function and dysfunction of the immune system in cancer. Ann. Oncol..

[101] Godfrey D.I., MacDonald H.R., Kronenberg M., Smyth M.J., Van Kaer L. (2004). NKT cells: What’s in a name?. Nat. Rev. Immunol..

[102] Terabe M., Swann J., Ambrosino E., Sinha P., Takaku S., Hayakawa Y., Godfrey D.I., Ostrand-Rosenberg S., Smyth M.J., Berzofsky J.A. (2005). A nonclassical non-Vα14Jα18 CD1d-restricted (type II) NKT cell is sufficient for down-regulation of tumor immunosurveillance. J. Exp. Med..

[103] Motohashi S., Nagato K., Kunii N., Yamamoto H., Yamasaki K., Okita K., Hanaoka H., Shimizu N., Suzuki M., Yoshino I. (2009). A phase I–II study of α-galactosylceramide-pulsed IL-2/GM-CSF-cultured peripheral blood mononuclear cells in patients with advanced and recurrent non-small cell lung cancer. J. Immunol..

[104] O’Neal J., Mavers M., Jayasinghe R.G., DiPersio J.F. (2024). Traversing the bench to bedside journey for iNKT cell therapies. Front. Immunol..

[105] Ramos C.A., Courtney A.N., Lulla P.D., Hill L.C., Kamble R.T., Carrum G., Wang T., Di Pierro E.J., Brenner M.K., Heslop H.E. (2024). Off-the-Shelf CD19-Specific CAR-NKT Cells in Patients with Relapsed or Refractory B-Cell Malignancies. Transplant. Cell. Ther..

[106] Toura I., Kawano T., Akutsu Y., Nakayama T., Ochiai T., Taniguchi M. (1999). Cutting edge: Inhibition of experimental tumor metastasis by dendritic cells pulsed with α-galactosylceramide. J. Immunol..

[107] Delfanti G., Cortesi F., Perini A., Antonini G., Azzimonti L., de Lalla C., Garavaglia C., Squadrito M.L., Fedeli M., Consonni M. (2022). TCR-engineered iNKT cells induce robust antitumor response by dual targeting cancer and suppressive myeloid cells. Sci. Immunol..

[108] Lameris R., Ruben J.M., Iglesias-Guimarais V., de Jong M., Veth M., van de Bovenkamp F.S., de Weerdt I., Kater A.P., Zweegman S., Horbach S. (2023). A bispecific T cell engager recruits both type 1 NKT and Vγ9Vδ2-T cells for the treatment of CD1d-expressing hematological malignancies. Cell Rep. Med..

[109] Heczey A., Xu X., Courtney A.N., Tian G., Barragan G.A., Guo L., Amador C.M., Ghatwai N., Rathi P., Wood M.S. (2023). Anti-GD2 CAR-NKT cells in relapsed or refractory neuroblastoma: Updated phase 1 trial interim results. Nat. Med..

[110] Ishibashi F., Sakairi Y., Iwata T., Moriya Y., Mizobuchi T., Hoshino H., Yoshida S., Hanaoka H., Yoshino I., Motohashi S. (2020). A phase I study of loco-regional immunotherapy by transbronchial injection of α-galactosylceramide-pulsed antigen presenting cells in patients with lung cancer. Clin. Immunol..

[111] Liu Y., Wang G., Chai D., Dang Y., Zheng J., Li H. (2022). iNKT: A new avenue for CAR-based cancer immunotherapy. Transl. Oncol..

[112] Alizadeh D., Wong R.A., Gholamin S., Maker M., Aftabizadeh M., Yang X., Pecoraro J.R., Jeppson J.D., Wang D., Aguilar B. (2021). IFNγ Is Critical for CAR T Cell-Mediated Myeloid Activation and Induction of Endogenous Immunity. Cancer Discov..

[113] Globerson Levin A., Rivière I., Eshhar Z., Sadelain M. (2021). CAR T cells: Building on the CD19 paradigm. Eur. J. Immunol..

[114] Abdelmegeed H., Nakamura T., Harashima H. (2016). In vivo inverse correlation in the activation of natural killer T cells through dual-signal stimulation via a combination of α-galactosylceramide-loaded liposomes and interleukin-12. J. Pharm. Sci..

[115] Guevara M.L., Jilesen Z., Stojdl D., Persano S. (2019). Codelivery of mRNA with α-Galactosylceramide using a new lipopolyplex formulation induces a strong antitumor response upon intravenous administration. ACS Omega.

[116] Coelho-Dos-Reis J.G., Huang J., Tsao T., Pereira F.V., Funakoshi R., Nakajima H., Sugiyama H., Tsuji M. (2016). Co-administration of α-GalCer analog and TLR4 agonist induces robust CD8^+^ T-cell responses to PyCS protein and WT-1 antigen and activates memory-like effector NKT cells. Clin. Immunol..

[117] Ando T., Ito H., Ohtaki H., Seishima M. (2013). Toll-like receptor agonists and alpha-galactosylceramide synergistically enhance the production of interferon-gamma in murine splenocytes. Sci. Rep..

[118] Aspeslagh S., Li Y., Yu E.D., Pauwels N., Trappeniers M., Girardi E., Decruy T., Van Beneden K., Venken K., Drennan M. (2011). Galactose-modified iNKT cell agonists stabilized by an induced fit of CD1d prevent tumour metastasis. EMBO J..

[119] Niedzielska M., Chalmers A., Popis M.C., Altman-Sharoni E., Addis S., Beulen R., Rudqvist N.P., Chantzoura E., Purbhoo M.A., Chand D. (2025). CAR-iNKT cells: Redefining the frontiers of cellular immunotherapy. Front. Immunol..

[120] Li Y.R., Zhu Y., Fang Y., Lyu Z., Yang L. (2025). Emerging trends in clinical allogeneic CAR cell therapy. Med.

